# Type 2 Diabetes Mellitus and Asthma: Pathomechanisms of Their Association and Clinical Implications

**DOI:** 10.7759/cureus.36047

**Published:** 2023-03-12

**Authors:** Pulkita Uppal, Shaza A Mohammed, Shriya Rajashekar, Suganya Giri Ravindran, Meghana Kakarla, Musa Ausaja Gambo, Mustafa Yousri Salama, Nathalie Haidar Ismail, Pardis Tavalla, Pousette Hamid

**Affiliations:** 1 Internal Medicine, California Institute of Behavioral Neurosciences & Psychology, Fairfield, USA; 2 Research, California Institute of Behavioral Neurosciences & Psychology, Fairfield, USA; 3 Neurology, California Institute of Behavioral Neurosciences & Psychology, Fairfield, USA

**Keywords:** glycemic control, asthma exacerbation, diabetes mellitus type 2, diabetes, asthma

## Abstract

Type 2 diabetes mellitus (T2DM) and asthma are chronic illnesses concomitantly present in a significant percentage of the population. Their comorbidity is associated with poor disease control and lower quality of life, thus imposing a substantial medical and economic burden worldwide. This review investigates the association between asthma and T2DM, in terms of pathogenesis, clinical outcomes, and therapeutic opportunities. Our review found an increased risk of asthma among diabetics, and vice versa. Having diabetes and poor glycemic control is associated with an increased rate of asthma exacerbations and increased mortality among those hospitalized for asthma exacerbations. The mechanisms postulated for the diabetes-asthma association include chronic low-grade inflammation, obesity, hyperinsulinemia, and possibly diabetic pneumopathy. Usage of metformin, which is the first-line drug for type 2 diabetes, was found to be associated with a decreased asthma occurrence, asthma exacerbations, and asthma-related hospitalizations. Glucagon-like peptide 1 receptor agonists were also found to be associated with a lower occurrence of asthma exacerbations. Thiazolidinediones are also associated with lower rates of asthma exacerbations, but their clinical efficacy for the same was suggested to be limited. This literature review supports a partly causative association between asthma and diabetes. This comorbidity leads to poor patient compliance, worse disease outcomes, and poor quality of life. Thus, further studies are warranted to explore the prognostic implications, therapeutic opportunities, and specific clinical practice algorithms for patients with concurrent asthma and type 2 diabetes mellitus.

## Introduction and background

Diabetes mellitus (DM) is a chronic disease characterized by the inability to transport glucose inside the cells, famously described as ‘starvation in the midst of plenty’. Hyperglycemia in type 2 diabetes mellitus (T2DM) arises because of a combination of insulin resistance, inadequate production of insulin by the β cells of the pancreatic islets, and hyperglucagonemia. T2DM, earlier thought of as a disease of the elderly, is now becoming increasingly prevalent amongst the younger population as well. The prevalence of type 2 diabetes worldwide was a staggering 438 million, and it resulted in 66.3 million disability-adjusted life years (DALYs) in 2019 [1].

Asthma is a chronic lung disease of the obstructive type. It is characterized by reversible airway obstruction because of bronchial hyperresponsiveness due to smooth muscle cell contraction and increased mucus production by goblet cells. These occur in response to the paracrine effects of the inflammatory mediators released by mast cell degranulation. Episodic cough, dyspnea, and wheezing are the most common presenting symptoms. The underlying chronic inflammation eventually leads to airway remodeling. Though primarily a disease beginning in the pediatric age group, asthma also often gets diagnosed later in adulthood. The prevalence of asthma was 262 million in 2019 and it was responsible for 21.6 million DALYs in the same year [1]. The entire incremental cost of asthma to society has been calculated at $56 billion, making asthma a considerable medical and economic burden worldwide [2].

The devastating consequences of diabetes mellitus include microangiopathy and thus retinopathy and nephropathy, macrovascular complications contributing to coronary artery disease and peripheral vascular disease, and diabetic neuropathy. However, the lung as a target organ for diabetic injury and the influence of diabetes on pulmonary function in lung diseases such as asthma are a relatively neglected subject in research [3,4]. Similarly, asthmatics have been found to be at an increased risk of suffering from diabetes mellitus, possibly because of underlying chronic inflammation, but a definitive causative role has not been successfully established [5].

Type 2 diabetes mellitus is a widely prevalent concomitant disease among adult asthma patients. Research has revealed the prevalence of comorbid diabetes and asthma to be as high as 16% [6]. Rates of health services claims for diabetes among individuals with asthma have been found to be higher than those for non-asthmatics [7]. Comorbid diabetes has been linked to poor asthma control, increased healthcare utilization, and a lower quality of life, and treating it has been proven to improve these outcomes significantly [8]. Understanding the influence of diabetes on asthma and vice versa is critical for better disease management and health outcomes of patients who have both these chronic diseases concurrently.

For the purpose of this review, the keywords ‘diabetes mellitus type 2’ and ‘asthma’ were used to search PubMed and Google Scholar to find the literature pertaining to the topic, and filters for full free texts and studies from the past 15 years were applied. This review makes an attempt to get a deeper insight into the pathophysiology of the diabetes-asthma association, and understand their mutual effect in terms of prognosis and clinical outcomes. Furthermore, we aim to explore evidence pointing to their therapeutic association and its potential implications in clinical practice guidelines.

## Review

Epidemiology

It is estimated that 34.1 million individuals aged 18 years or older in the United States (US), equivalent to 13.0% of the national adult population, were suffering from diabetes mellitus in 2018. This number for pre-diabetes, defined as hyperglycemia that does not meet the criteria for an outright diabetes diagnosis, was a staggering 88 million, i.e. 33.5% of all US adults [9]. In 2018, 24.8 million persons in the US, i.e. 7.7% of the population, had asthma; asthma exacerbations were responsible for 1.6 million emergency department visits and 183,000 hospitalizations in the US in 2017 [10]. The prevalence of concurrent asthma and diabetes has been found to be as high as 16%, and thus, the healthcare burden of the multimorbidity of these chronic diseases is massive [6].

A study found that the incidence of asthma was higher in the diabetic cohort than the non-diabetic group after adjusting for age, sex, and obesity, with a hazard ratio (HR) of 1.30 (95% confidence interval, or CI, 1.24-1.38) [11]. In a cross-sectional study conducted among 5045 Korean adults, it was discovered that the probability of asthma occurrence in the DM-diagnosed group was 1.75 times higher than the non-asthma group (95% CI 1.06-3.02; p = 0.048). In the same study, the authors found that the risk of occurrence of asthma was 1.38 times greater in people with high glycated hemoglobin (HbA1c) levels (95% CI 1.03-1.84; p = 0.031) and 1.02 times higher in people with higher insulin levels (95% CI 1.01-1.04; p = 0.015) [12].

A prospective cohort study including 38,570 women concluded that having asthma was related to an increased risk of T2DM. On investigating for potential effect modifiers such as age, smoking, body mass index (BMI), and alcohol consumption, the positive association was slightly attenuated but did still exist. These findings insinuate the role of chronic inflammation in the pathogenesis of the asthma-diabetes comorbidity [13]. The Study to Help Improve Early evaluation and management of risk factors Leading to Diabetes (SHIELD) found that persons with asthma had a 33% higher chance of transitioning to type 2 diabetes (p = 0.020). This indicates that asthma is one of the predictors of its development. Other factors found to be predictive in the same study were age, a positive family history, obesity, visceral fat, and symptoms such as increased thirst [5].

Effect of diabetes on the clinical course of asthma

A prospective study concluded that hyperglycemia is significantly associated with the risk of an extended hospitalization due to asthma exacerbation, regardless of the route of insulin administration. It was found that the average hospitalization time was 8.2 days (insulin infusion) and 10.4 days (subcutaneous insulin) in those with hyperglycemia. This duration for the admitted patients without any glucose metabolism derangement was only 5.2 days [14].

A study of 5722 individuals found that compared to individuals with HbA1c in the normal range (HbA1c <5.7%), those in the pre-diabetes (5.7%-6.4%) and diabetes (>6.5%) categories had a 27% and 33% greater asthma exacerbation rate, respectively (p = 0.01). In the continuous model, for the most part, the relationship between HbA1c and asthma exacerbation rate was linear. A 0.5% increase in HbA1c was linked to a 14% increase in the asthma exacerbation rate in the linear part of the curve (95% CI 4%-25%) [15]. A large cross-sectional study of 47,606 adults found that people with an HbA1c level in the pre-diabetes or diabetes range had a 1.68 times higher risk of one asthma-related hospitalization than people with a normal HbA1c level (p = 0.01). In the same study, the multivariable analysis showed that a 1% increase in HbA1c was associated with a 3% increase in the likelihood of one asthma-related hospitalization (p = 0.01) [16].

Research has shown that a diagnosis of diabetes is strongly associated with increased mortality in patients hospitalized for asthma exacerbations (adjusted HR = 3.03, CI 1.28-7.18). The same was not observed amongst those who had hyperglycemia during the exacerbation, but were not otherwise diabetic [17]. A Taiwanese study identified diabetes mellitus as an independent risk factor for mortality in patients hospitalized due to asthma exacerbation, amongst others such as pneumonia, septicemia, use of short-acting β2 agonists (SABAs), and oral corticosteroid use of more than 110 mg prednisolone per month in outpatient treatment [18].

Pathomechanisms of the association between asthma and T2DM

Diabetes-Lung Association and Its Implication for Asthma

Diabetes mellitus is a multi-systemic disease that affects many organs of the body. Diabetic retinopathy, nephropathy leading to end-stage renal disease, neuropathy, and macroangiopathy leading to cardiovascular disease and non-traumatic limb amputations are well-established complications of diabetes. The lung as a target organ for diabetic injury is however a relatively less explored niche, and ‘diabetic pneumopathy’ is not a known entity in the medical literature [3]. It has been found that when compared to the general population of the same age, people with type 2 diabetes have a higher prevalence of respiratory symptoms, such as dyspnea, chronic cough, and phlegm. This supports that diabetes may anticipate the pulmonary aging process [19].

Non-enzymatic reactions between glucose precursors and amino group of proteins lead to the formation of advanced glycation end-products (AGEs), which bind to the receptor for AGE (RAGE). AGE-RAGE signaling leads to expression of cytokines, growth factors, proliferation of vascular smooth muscle cells, and synthesis of extracellular matrix. RAGE signaling is abundantly expressed in the lungs and has been implicated as a mechanism for diabetes-associated chronic airway and vascular inflammation [20].

**Figure 1:**
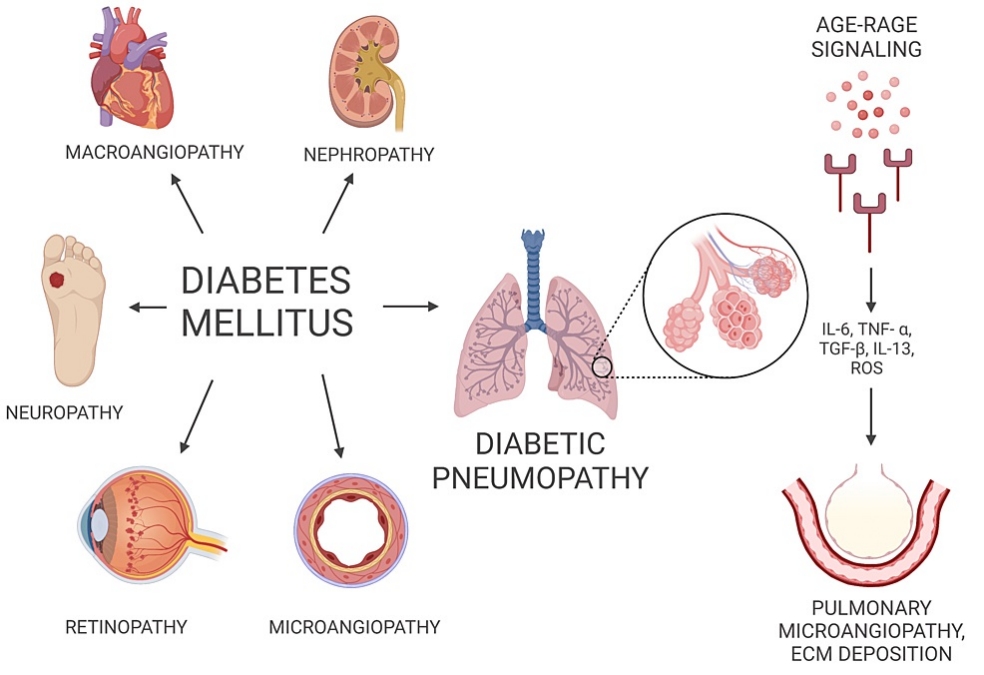
Multi-organ effect of diabetes on the body AGE: advanced glycation end-products, RAGE: receptor for advanced glycation end-products, IL: interleukin, TNF-α: tumor necrosis factor α, TGF-β: transforming growth factor, ROS: reactive oxygen species, ECM: extra-cellular matrix Image credits: Created by authors on BioRender software (BioRender, Toronto)

The lung contains a complex alveolar-capillary network that can be targeted by diabetic microvascular injury and lead to a decline in lung function in pulmonary diseases, including asthma [21,22]. A nationwide Korean study showed that diabetic individuals with retinopathy had a higher risk of developing asthma, further implying that the pulmonary vascular network may be the target of T2DM-related microangiopathy [23]. Other mechanisms include hyperglycemia-induced production of matrix metalloproteinase 9 which could lead to airway epithelial barrier dysfunction and inflammation [24]. Figure 1 represents the effects of diabetes on various organs of the body.

Inflammation

Low-grade inflammation, as measured by higher levels of interleukin-6 (IL-6), tumor necrosis factor (TNF), C-reactive protein (CRP), and adhesion molecules, has been identified as a major contributor to the development of T2DM [25]. Asthma is thought to be caused by the nuclear factor-B (NF-κB) signaling pathway [26]. NF-κB is a transcription factor that regulates the expression of IL-6, TNF, and adhesion molecules, among other pro-inflammatory genes. It seems reasonable to speculate that increased circulating levels of certain inflammatory cytokines caused by chronic airway inflammation may contribute to the development of insulin resistance in the liver, skeletal muscle, and vascular endothelium, eventually leading to the clinical manifestation of diabetes. Obesity is a chronic disease with underlying low-grade inflammation, both of which may interact to accentuate diabetes development, but inflammation has been proven to contribute regardless of the BMI as well [13].

Participants with asthma and non-asthmatic controls were compared in a retrospective cohort research to see how likely they were to acquire proinflammatory illnesses such as type 2 diabetes mellitus and coronary heart disease (CHD). The authors found an increased risk of T2DM (HR = 2.12, 95% CI 1.43-3.14, p > 0.001) and CHD (HR = 1.50, 95% CI 1.07-2.10, p = 0.02) in asthma. They claimed that the link between asthma-T2DM and asthma-CHD is likely due to the shared immunogenic and environmental factors, such as the production of inflammatory cytokines IL-6 and IL-17, which have been found in both of these diseases [27].

Obesity

Obesity is associated with low-grade systemic inflammation and increased levels of inflammatory markers such as the toll-like receptor 4 (TLR4), tumor necrosis factor-alpha (TNF-α), and IL-6 [28].

Obesity is a known contributor to asthma occurrence, as confirmed by a study that found that all obesity measurements and insulin resistance were linked to wheezing and asthma-like symptoms. This discovery is backed by the idea that obesity and asthma are related through inflammatory pathways that are also involved in insulin resistance [29]. Obesity is a known risk factor for diabetes as well, because of insulin resistance and underlying low-grade inflammation [5,30]. Asthma and obesity may work in a synergistic manner to raise the circulating levels of proinflammatory cytokines, increasing the risk of insulin resistance and T2DM. A hyperinflammatory state could be the most important etiology driving the development of both asthma and obesity. As a result, the two conditions might sustain one other via vicious reinforcing proinflammatory pathways that eventually impair glucose metabolism.

A prospective cohort study conducted among Singaporean Chinese adults reported that the participants with a previous diagnosis of asthma had a 31% higher risk of developing T2DM. The association was stronger for participants who were obese than those who were not, suggesting that having more body fat and inflammation could interact to contribute to diabetes development [31]. Positive genetic associations were discovered in a Danish twin study between asthma and type 2 diabetes, asthma and BMI in women, and BMI and type 2 diabetes. This supports the role of obesity in the manifestation of both DM and asthma [30].

Insulin Resistance

Insulin resistance precedes hyperglycemia and leads to compensatory hyperinsulinemia in the early stages of diabetes. It has not only been found to be associated with asthma in diabetics [32], but is also postulated to play a causal role as shown in an animal study [33]. In a cross-sectional study, the mean insulin levels were found to be 10.19 and 8.43 mU/L for the asthmatic and non-asthmatic groups, respectively (p = 0.010). Additionally, it was discovered that the occurrence of asthma was 1.02 times higher in people with higher insulin levels (95% CI 1.01-1.04; p = 0.015) [12]. A cross-sectional study further confirmed the linkage between insulin resistance and asthma in patients with T2DM. It showed a significant association of asthma with homeostasis model assessment (HOMA) index and visceral fat accumulation (OR = 3.65; 95% CI 1.37-7.85, and OR = 1.78; 95% CI 1.31-3.89, respectively) [32].

**Figure 2:**
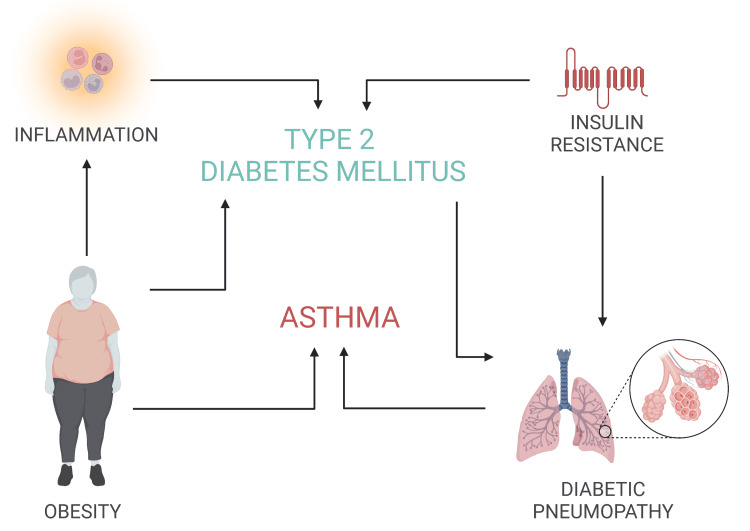
Factors responsible for the association between asthma and type 2 diabetes mellitus Image credits: Created by authors on BioRender software

A study showed that treatment with inhaled insulin was responsible for increased airway hyperresponsiveness, human smooth muscle proliferation, collagen deposition, and peri-bronchial thickening (p < 0.05), which are similar to the pulmonary findings in asthma [22]. Hyperinsulinemia has also been proven to potentiate vagally induced bronchoconstriction by lowering the activity of inhibitory M2 muscarinic receptors on parasympathetic neurons in the absence of inflammation, resulting in increased acetylcholine release and airway hyperresponsiveness, as measured in rats [33]. Other possible pathways by which hyperinsulinemia may also compromise pulmonary function include lowering the bioavailability of the endogenous bronchodilator nitric oxide or by causing fibroblast proliferation [22,34]. Figure 2 summarizes the pathomechanisms responsible for the association between asthma and type 2 diabetes mellitus.

Diabetes treatment and asthma

It is imperative to focus on formulating patient-centered treatment plans for those suffering from multimorbidity, such as concurrent asthma and diabetes mellitus. Increased treatment load can be overwhelming for the individual and lead to poor patient compliance, and worsen disease control and prognosis [35]. Moreover, it has been found that in the case of comorbidity of asthma and diabetes, the prescribed treatment might be insufficient to achieve adequate control because of the underlying pathophysiology of the diseases, and a mere increase in the dosage of corticosteroids is not the solution, because of the adverse effects on glycemic control [36]. Thus, it is important to explore how the treating drugs affect disease control and to look for potentially improved therapeutic approaches.

Metformin

Metformin use was linked to a lower incidence of asthma, according to a retrospective cohort analysis (OR 0.75, 95% CI 0.60-0.95). In contrast, insulin was discovered to raise the incidence of asthma in diabetic patients in the same study [11]. A meta-analysis found that metformin use was associated with a reduced risk of asthma-related emergency department visits amongst patients with concurrent asthma and diabetes (OR = 0.81, 95% CI 0.74-0.89). A decrease in the rates of asthma exacerbations and asthma-related hospitalizations was also observed; however, it was not statistically significant [37].

A retrospective cohort study found that metformin users had a decreased risk of asthma-related hospitalization (OR = 0.21; 95% CI 0.07-0.63) and asthma exacerbation (OR = 0.39; 95% CI 0.19-0.79) than metformin non-users [38]. A cohort study consisting of 23,920 individuals discovered that metformin treatment was linked to fewer asthma-related emergency department visits (HR 0.81; 95% CI 0.74-0.88) and hospitalizations (HR 0.67; 95% CI 0.50-0.91) [39].

One of the mechanisms for the above-mentioned findings could be the stimulation of adenosine monophosphate-activated protein kinase (AMPK) by metformin, which is also responsible for its effect on insulin sensitivity. By activation of AMPK, it inhibits glycolysis and cytokine production in immune cells in vitro and in vivo [40]. Metformin also attenuates airway inflammation, fibrosis, and remodeling by activating AMP-activated protein kinases and inhibiting mTOR signaling [41]. It could be affecting asthma-related clinical outcomes due to amelioration of insulin resistance and aiding in weight loss as well, as both insulin resistance and obesity are known to contribute to asthma [29,32].

Glucagon-Like Peptide 1 Receptor Agonists

Glucagon-like peptide 1 receptor (GLP1R) agonists act by mimicking incretin, thus increasing insulin secretion and reducing glucagon release in response to glucose [42]. It has been found that the occurrence of asthma exacerbations was lower in patients receiving GLP1R agonists (i.e. reference) compared to those on sodium glucose transporter 2 (SGLT-2) inhibitors (incidence rate ratio, or IRR, 2.98; 95% CI 1.30-6.80), dipeptidyl peptidase-4 (DPP-4) inhibitors (IRR 2.45; 95% CI 1.54-3.89), sulfonylureas (IRR 1.83; 95% CI 1.20-2.77), and basal insulin (IRR 2.58; 95 % CI 1.72-3.88). The findings were robust to adjustment for BMI or HbA1C, implying that the effects were independent of weight or glycemic control [43].

GLP1R agonists activate the cyclic AMP-dependent protein kinase A in the human airway and relax airway smooth muscle cells, thus proving GLP1 receptors to be potential targets for treating airway hyperresponsiveness [44]. Similar to the effect induced by β2-agonists, GLP1 analogs produce a synergistic broncho-relaxant effect on airway smooth muscle cells when used in combination with other bronchodilating agents with different mechanisms of action, such as antimuscarinic drugs. This strategy could allow for the reduction of individual agent doses and help avoid the danger of adverse effects from either drug provided alone at higher concentrations [44,45].

Thiazolidinediones

Thiazolidinediones are oral antidiabetic drugs acting on peroxisome proliferator-activated receptor gamma (PPARγ), and they have been shown to effectively decrease bronchial hyperresponsiveness, airway inflammation, and levels of Th2 cytokines [46].

A small pilot study suggested limited efficacy of pioglitazone in the treatment of poorly controlled asthma in obesity and highlighted weight gain as its adverse effect [47]. On the other hand, exposure to thiazolidinediones was linked with significant reductions in the risk of asthma exacerbation (OR = 0.79, 95% CI 0.62-0.99) and oral steroid prescription (OR = 0.73, 95% CI 0.63-0.84) in a cohort study of 13,528 participants [48].

Insulin

Insulin therapy is employed in T2DM when oral antidiabetic drugs fail to adequately control blood glucose levels after multiple glucose-lowering agents have been initiated in a step-wise manner during the progressive course of the disease [49].

In vitro and animal studies have shown that increased insulin levels are associated with higher bronchial smooth muscle proliferation and airway hyperresponsiveness [22,33]. A study conducted in a Taiwanese population cohort discovered that the use of insulin therapy amongst diabetics was associated with a higher occurrence of asthma (OR = 2.23; 95% CI 1.52-3.58) [11].

Limitations

The limitations of this review could be attributed to the possible confounding bias caused due to antidiabetic medication, when comparing clinical outcomes of asthma in diabetics. Similarly, there might be reverse causation when linking glycemic control to asthma exacerbations, where poor glycemic control might be because of increased corticosteroid use due to poorly controlled asthma, and not vice versa [15,16]. Another limitation is that this narrative review has not excluded animal studies, even though they are few in number.

## Conclusions

From this literature review, it is abundantly clear that there is a significant association between the occurrence of asthma and type 2 diabetes mellitus, the likely pathomechanisms for which include pulmonary microangiopathy, low-grade inflammation, obesity, and insulin resistance. Unfortunately, this means that each of these disorders imposes extra health burdens on top of those known already. The review also revealed that the use of antidiabetic drugs, notably metformin, and GLP1 receptor agonists is linked to a decreased occurrence of asthma exacerbations among diabetics.

Comorbidity of asthma with diabetes worsens clinical outcomes and leads to increased healthcare burden. Asthmatics tend to have poor glycemic control due to a multitude of factors. At the same time, diabetics who have asthma show more obstruction manifestations, exacerbations requiring hospitalization, and shorter spirometric remissions, not to mention the possibility of ‘diabetic pneumopathy’. The excessive treatment burden in these patients with multimorbidity can lead to poor compliance, worsening clinical outcomes even further. As a result, distinct patient-centered clinical guidelines and novel therapeutic approaches are required for such a subset of patients. Thus, the exploration of prognostic and therapeutic implications of the association between asthma and diabetes can pave the way for the development of better clinical practice algorithms, which would improve the quality of life for patients in whom both these chronic diseases are co-existent.
